# Resolving the TorsinA Oligomerization Conundrum: The Glycan Hypothesis

**DOI:** 10.3389/fmolb.2020.585643

**Published:** 2020-10-02

**Authors:** Christian Fercher, Lucía F. Zacchi

**Affiliations:** Australian Research Council (ARC), Training Centre for Biopharmaceutical Innovation, The University of Queensland, St Lucia, QLD, Australia

**Keywords:** torsinA, glycans, AAA ATPase, modeling, structure

## Abstract

TorsinA is a AAA+ ATPase involved in the severe neurological disease Early Onset Torsion Dystonia. Despite the impressive progress in the field in the recent years, the structural organization and function of this intriguing molecule is still not clear. One outstanding difference between torsinA and other AAA+ ATPases is that torsinA is a glycoprotein. TorsinA *N*-linked glycans impact torsinA biogenesis and subcellular localization. Here, we propose that torsinA glycans also modulate torsinA oligomerization properties. We used structural modeling to test this idea, and show that *N*-linked glycans appear to restrict torsinA’s ability to form closed homohexameric ring assemblies, and instead promote an open hexameric conformation that allows torsinA interaction with key cofactors required for ATP hydrolysis. This mechanism would make torsinA a prime example of Nature’s sophisticated molecular glycoengineering.

## Introduction

TorsinA is an Endoplasmic Reticulum (ER) AAA+ ATPase associated with the development of the neurological disease Early-Onset Torsion Dystonia (EOTD) (Gonzalez-Alegre, 2019). TorsinA structure and function, and its role in EOTD onset are still controversial (Rose et al., 2015; Chase et al., 2017a). TorsinA is a rather unusual ATPase, as it lacks the AAA+ ATPase conserved hydrophobic pore loops involved in substrate unfolding and remodeling and the arginine finger required for ATP hydrolysis (Brown et al., 2014; Sosa et al., 2014; Demircioglu et al., 2016). In fact, the ATP hydrolysis activity of torsinA requires binding to the ER and Nuclear Envelope-type II transmembrane proteins LULL1 and LAP1, respectively (Zhao et al., 2013; Brown et al., 2014; Sosa et al., 2014). Further, AAA+ ATPases generally adopt hexameric conformations (Hanson and Whiteheart, 2005), but torsinA oligomerization status is still under debate. TorsinA has been proposed to form a homohexameric structure, and/or a heterohexameric structure with LULL1/LAP1, and/or a homopolymeric filament structure (Vander Heyden et al., 2009; Jungwirth et al., 2010; Zhao et al., 2013; Brown et al., 2014; Li et al., 2014; Sosa et al., 2014; Demircioglu et al., 2016, 2019; Chase et al., 2017a,b). It is unclear whether any of these oligomers reflect the physiological conformation of torsinA, and if torsinA can dynamically transition from one to another.

**FIGURE 1:**
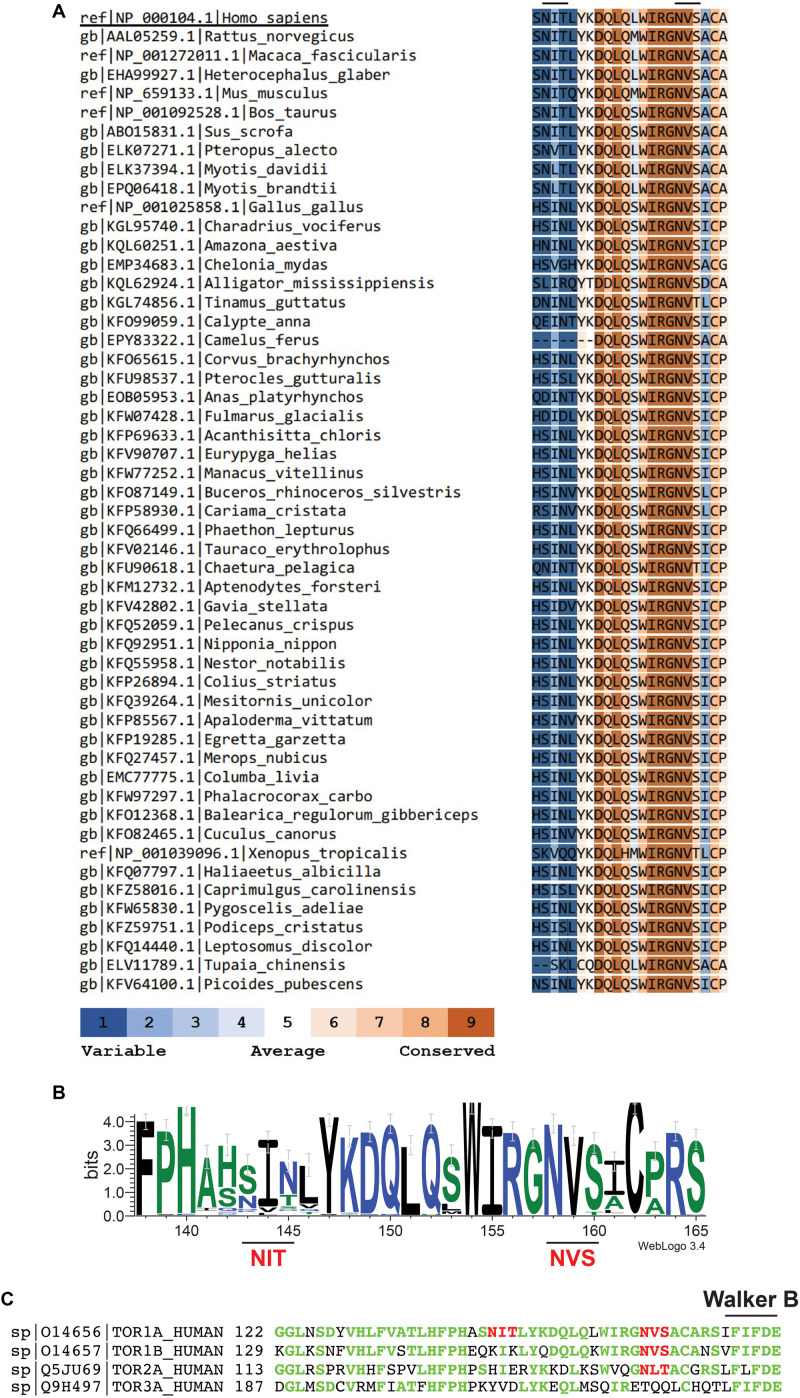
The second *N*-linked glycosylation site, but not the first, is conserved in torsinA homologs. **(A)** Human torsinA was aligned to 51 torsinA amino acid sequences spanning multiple species. Conservation scores were calculated using the ConSurf web server (https://consurf.tau.ac.il/). **(B)** Sequence logo showing the conservation of the torsinA amino acid region containing the N^143^IT and N^158^VS *N*-linked glycosylation sites. The graphical comparison was obtained using Weblogo (http://weblogo.berkeley.edu/) (Crooks et al., 2004). **(C)** Alignment of human torsinA amino acid sequence with its homologs torsin1B, torsin2, and torsin3 (Uniprot identifiers and starting amino acid number are shown to the left of the sequences). The N^143^IT and N^158^VS *N*-linked glycosylation sites are highlighted in red and the Walker B ATP hydrolysis domain is indicated in black. The torsinA amino acids that are conserved in the homologs are shown in green.

Critically, the proposed polymeric structures for torsinA do not take into account one fundamental molecular characteristic of torsinA: that torsinA is a glycoprotein. TorsinA has two sites for *N*-linked glycosylation located between the ATP binding and hydrolysis Walker domains (Ozelius et al., 1997; Figure 1). Both *N*-linked glycosylation sites are occupied in torsinA (Hewett et al., 2000, 2003; Kustedjo et al., 2000; Bragg et al., 2004; Gonzalez-Alegre and Paulson, 2004; Vander Heyden et al., 2011; Zacchi et al., 2014; Zhao et al., 2016). The N^158^VS *N*-linked glycosylation site is highly conserved in torsinA homologs across different species (Figures 1A,B) and in the other three torsinA human homologs (Figure 1C; Ozelius et al., 1999; Zhu et al., 2008; Granata et al., 2009; Demircioglu et al., 2016), while the N^143^IT glycosylation site is less conserved (Figure 1). *N*-linked glycans are heavily involved in protein folding and quality control (Aebi, 2013; Caramelo and Parodi, 2015; Zacchi et al., 2016). Indeed, torsinA requires at least one *N*-linked glycan for stability (Hewett et al., 2003; Zacchi et al., 2014). Interestingly, glycosylation at N^158^VS is selectively required to stabilize the EOTD-associated torsinAΔE variant (Zacchi et al., 2014) and mutation of this site reverts the aberrant torsinAΔE subcellular localization (Bragg et al., 2004). Therefore, *N*-linked glycans are key post-translational modifications for torsinA biogenesis. *N*-linked glycans are bulky and hydrophilic molecules (Varki, 2017) and, in addition to their role in folding and quality control, *N*-linked glycans can restrict the formation of quaternary structures in proteins (Medus et al., 2017). This conformation-shaping effect has been well documented for immunoglobulin G (Nagae and Yamaguchi, 2012; Higel et al., 2016). Hence, *N*-linked glycans could also influence torsinA ability to form hexamers or filaments and interact with membranes and other proteins, impacting torsinA function.

## Results and Discussion

**FIGURE 2:**
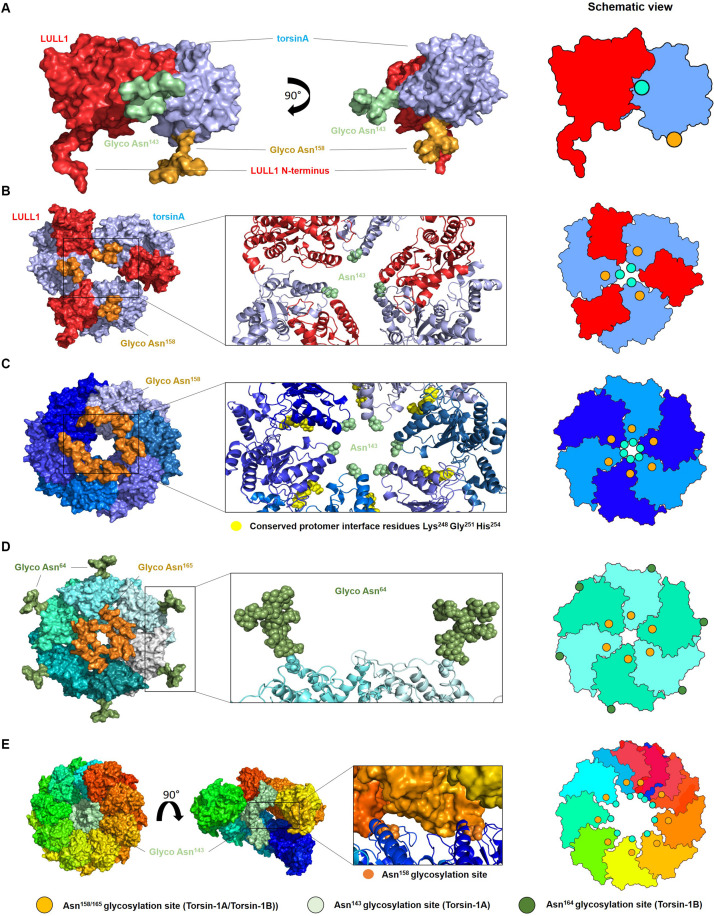
Oligomerization of glycosylated torsinA. **(A)** Typical ER high mannose glycans were attached to Asn^143^ (green) and Asn^158^ (orange) of torsinA (blue) using the high-resolution structure of the protein in complex with the C-terminal domain of its native interaction partner LULL1 (red) (PDB ID 5J1S) (Demircioglu et al., 2016). The N-terminal residues preceding LULL1 TMD are visible in the crystal structure. **(B)** Predicted heterohexameric model of glycosylated torsinA:LULL1 complex with the LULL1 N-terminus and Asn^158^ *N*-linked glycans potentially facing the ER membrane. Generation of this model was only possible in the absence of Asn^143^ *N*-linked glycans as these face the central opening of the ring which is not wide enough to accommodate three chains simultaneously. **(C)** Extended torsinA hexamer conformation showing the position of the Asn^158^ glycan. The amino acids involved in the conserved protomer interface are highlighted in yellow (Chase et al., 2017b). **(D)** Homohexameric glycosylated torsinB. *N*-linked glycans at Asn^64^ and Asn^165^ (equivalent to Asn^158^ in torsinA) are shown in green and orange, respectively. **(E)** Exemplary segment of a torsinA filament (PDB ID 6OIF) in rainbow colors to indicate individual monomers. The middle structure is presented in open side view to illustrate the internal arrangement of the Asn^143^ *N*-linked glycan chains. The position of two consecutive Asn^158^ glycosylation sites between individual turns of the filament are indicated as orange spheres. Structures were modeled as follows. TorsinA and torsinB (Uniprot ID O14656 and O14657) were *in silico* (*glycosylated with high mannose *N*-linked glycans using CHARMM-GUI Glycan Modeler (Park et al., 2019). Three copies of the torsinA-LULL1 glycoprotein complex were used to construct a heterohexameric model based on the closed conformations of Hsp104 (6N8T) and ClpC (3PXI), which represent the closest torsinA homologs in terms of sequence identity for which hexameric ring structures are currently available. Both complexes were virtually identical and those based on 6N8T were subsequently used for all models. Structures were energy minimized to resolve minor sterical clashes at the subunit interfaces and to create glycan conformations compatible with a hexameric assembly employing the YASARA force field (Krieger et al., 2009). The homology model of torsinB was generated with Modeler (Webb and Sali, 2016) using torsinA as a template (67% sequence identity). Glycosylation and complex building were done analogous to torsinA. Alignments and figures were prepared using PyMOL (Schrödinger). Schematic views of the oligomers are shown on the right, with the green and orange circles indicating the position of the Asn^143^ and Asn^158^*N*-linked glycans, respectively.*)

**FIGURE 3:**
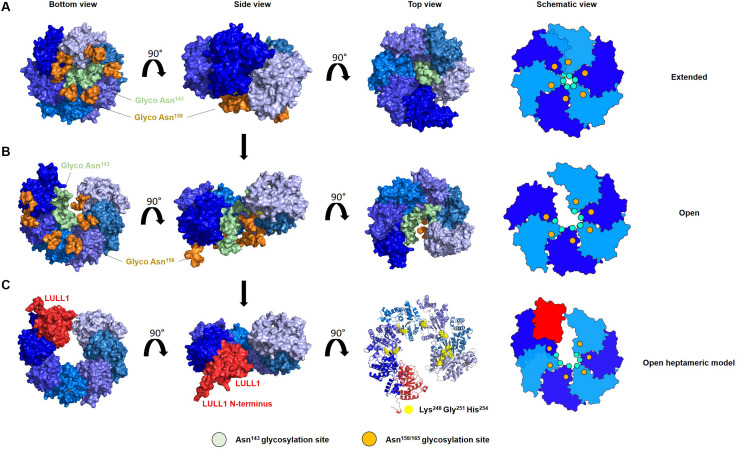
*N*-linked glycosylation of torsinA may promote formation of an open hexameric ring structure *in vivo*. Proposed model of the transition of glycosylated torsinA from extended **(A)** to open homohexameric conformation **(B)** and interaction with LULL1 cofactor (shown in red) **(C)**. TorsinA is colored in varying shades of blue to facilitate identification of individual protein subunits, and is modeled according to PDB 6N8Z and 6N8V as templates (Hsp104 extended and open conformation, respectively). High mannose glycans were attached to Asn^143^ (green) and Asn^158^ (orange). **(A–C)** The torsinA-LULL1 structure was fitted in the penultimate position of the open ring structure to generate a model of a potentially transient heptameric complex. For clarity, glycans were omitted in this representation but would not interfere with binding of LULL1. Conserved residues which are crucial for complex assembly are depicted as yellow spheres. Schematic views of the oligomers are shown on the right, with the green and orange circles indicating the position of the Asn^143^ and Asn^158^ *N*-linked glycans, respectively.

Modeling diverse oligomerization states of glycosylated torsinA supports the intriguing hypothesis that *N*-linked glycans play a fundamental structural and functional role in torsinA (Figure 2). To generate these models, we used the torsinA-LULL1 heterodimer crystal structure (PDB 5J1S) (Demircioglu et al., 2016). The torsinA-LULL1 structure displays torsinA without the transmembrane domain (TMD) and the ER lumenal (C-terminal) domain of LULL1 with the N-terminal residues that follow the TMD (Demircioglu et al., 2016). We modeled the position of the *N*-linked glycans in torsinA based on this structure by attaching typical ER high mannose *N*-linked glycans at Asn^143^ and Asn^158^ (Hewett et al., 2000; Kustedjo et al., 2000; Aebi, 2013; Figure 2). In this model, the Asn^158^ glycan would be oriented parallel to the TMD, potentially facing the ER membrane while the Asn^143^ glycan would protrude from torsinA in a close to 90° angle (Figure 2A). Due to their position in the folded protein, torsinA *N*-linked glycans would not impact heterodimerization with the LULL1/LAP1 cofactors (Figure 2A), as expected for this physiological interaction. However, structural modeling suggests that the Asn^143^ glycan would prevent homo- and heterohexameric closed ring assemblies, as these glycans appear to face the constricted space of the central opening of the ring (Figures 2B,C, 3). Interestingly, this glycomolecular crowding would not be an issue for torsinB, torsinA’s paralog (Ozelius et al., 1999; Hewett et al., 2004), since the N^143^IT glycosylation site is not conserved in torsinB (Figure 1C). Instead, torsinB contains a glycosylation site at Asn^64^, which is not oriented towards the inner ring but rather towards the outer perimeter (Figure 2D), potentially allowing for the formation of closed torsinB homohexameric rings (Figure 2D). The molecular crowding of Asn^143^ *N*-linked glycans in this region of torsinA can be resolved when modeling torsinA homohexamers based on extended (Figures 2C, 3A) and open (Figure 3B) structural conformations of the homologous AAA ATPase Hsp104 (Lee et al., 2019). In these scenarios, the respective glycans would be accommodated by a slightly larger central opening and a pitch that results from a lock-washer-like conformation moving individual Asn^143^ residues out of plane. Similarly, glycan chains at Asn^143^ could be seamlessly accommodated by the larger central pore of torsinA filaments (Demircioglu et al., 2019; Figure 2E). However, Asn^158^ glycans would most likely prevent further polymerization due to geometrical restrictions that arise from interactions of torsinA molecules in consecutive turns of the ring which essentially block access to Asn^158^ (Figure 2E). Therefore, the presence of *N*-linked glycosylation on Asn^143^ and Asn^158^ may promote assembly of torsinA into the proposed open homohexameric ring structures *in vivo* (Chase et al., 2017a,b). Intriguingly, this model would provide a basis to reconcile observations of homohexameric torsinA complexes with the fact that monomers need to be accessible for binding LULL1/LAP1 cofactors to exert their enzymatic function (Li et al., 2014; Chase et al., 2017a,b; Figure 3C). Based on our observations, we propose that fully glycosylated torsinA can only assemble into extended (Figure 3A) or open (Figure 3B) hexameric structures upon binding ATP (Chase et al., 2017b). These complexes would be stabilized by the presence of the Asn^143^ and Asn^158^ glycans that prohibit closed ring structures or further polymerization into filaments. The transition between extended and open forms may be dynamic or could be triggered by interactions with the torsinA cofactor LULL1/LAP1 but, ultimately, an open conformation would be required to accommodate the torsinA-LULL1 heterodimer at the penultimate position of the complex (see Figure 3C for a model of the proposed transient heteroheptameric torsinA-LULL1 complex) (Chase et al., 2017a,b). Binding of the cofactor results in ATP hydrolysis, concomitant with ring dissassembly and loss of the torsinA subunit (Chase et al., 2017b).

Notably, glycosylation is not required for torsinA oligomerization, as bacterially expressed torsinA can assemble into hexamers and filaments (Sosa et al., 2014; Demircioglu et al., 2016, 2019) [although important caveats to the bacterial expression system that question the physiological relevance of these structures have been pointed out (Chase et al., 2017b; Demircioglu et al., 2019)]. Further, open ring conformations are naturally adopted by multiple other (cytosolic) AAA+ ATPases (Dong et al., 2019; Lee et al., 2019; Gates and Martin, 2020; Lopez et al., 2020), indicating that *N*-linked glycosylation is not required to generate open ring conformations. However, when present, torsinA *N*-linked glycans would ensure that this unusual ATPase maintains the open/extended oligomeric conformation required for LAP1/LULL1-dependent ATP hydrolysis by preventing the formation of closed ring assemblies. Therefore, torsinA *N*-linked glycans would play an essential role in modulating torsinA oligomerization and function.

The potential impact of *N*-linked glycans on torsinA oligomerization would add another layer of complexity into the regulation of torsinA function. In this context, genetic or environmental factors that lead to defects in protein translocation into the ER, in protein glycosylation, or in glycan-dependent folding or quality control would impact torsinA ability to form closed or open hexamers and, in turn, its ability to interact with its cofactors. Considering the delicate equilibrium that governs ER homeostasis, how torsinA *N*-linked glycosylation is sensitive to redox changes and to defects in certain ER chaperones and enzymes (Zacchi et al., 2014), and how accessibility to these enzymes and chaperones could vary throughout cell development or due to cell type or subcompartment localization (Shenkman and Lederkremer, 2019; Almeida and Amaral, 2020), it is tempting to speculate that *N*-linked glycans offer torsinA the key to a dynamic conformational and functional molecular polymorphism.

*N*-linked glycans are highly diverse structures that play myriad of functional roles in the cell (Varki, 2017). *N*-linked glycans are usually studied in the context of protein quality control during folding and biogenesis, of genetic diseases, and due to their role in immunological responses and microbial pathogenesis. However, it is becoming increasingly clear that the occupancy and structural heterogeneity of the glycans play fundamental structural and physiological roles (Higel et al., 2016; Zacchi and Schulz, 2016; Medus et al., 2017). Here, we highlight the key function of *N*-linked glycans in shaping the conformation and structure of protein complexes (Medus et al., 2017), and hypothesize that their presence or absence dramatically impact torsinA oligomerization by introducing sterical restrictions. The last decades have seen phenomenal advances in protein and glycan structural characterization technologies. The combination of both fields of studies will not only unravel the quaternary structure of this fascinating protein, but will also provide an exciting new magnifying glass into biology.

## Data Availability Statement

Publicly available datasets were analyzed in this study. This data can be found here: All datasets used are available in https://www.rcsb.org/. The specific datasets used in this work are: PDB ID 5J1S, PDB ID 6OIF, PDB ID 6N8T, PDB ID 3PXI, PDB ID 6N8Z, and PDB ID 6N8V.

## Author Contributions

CF and LZ were involved in all aspects of this work regarding conception and design, data analysis and interpretation, writing and editing of the manuscript, and made the figures. CF performed the structural modeling. Both authors contributed to the article and approved the submitted version.

## Conflict of Interest

The authors declare that the research was conducted in the absence of any commercial or financial relationships that could be construed as a potential conflict of interest.
